# Transcriptome analysis of the effect of diosgenin on autoimmune thyroiditis in a rat model

**DOI:** 10.1038/s41598-021-85822-1

**Published:** 2021-03-18

**Authors:** Chengfei Zhang, Lingling Qin, Boju Sun, You Wu, Fengying Zhong, Lili Wu, Tonghua Liu

**Affiliations:** 1 Dongfang Hospital of Beijing University of Chinese Medicine, Beijing, China; 2 Technology Department, Beijing University of Chinese Medicine, Beijing, China; 3 Key Laboratory of TCM Health Cultivation of Beijing, Beijing University of Chinese Medicine, Beijing, China

**Keywords:** Thyroid diseases, Thyroid diseases, Autoimmunity

## Abstract

In a mouse model of Graves’ disease (GD), diosgenin has been shown to have a therapeutic effect on GD by alleviating goitre. However, research on the effect of diosgenin on autoimmune thyroiditis (AIT) is lacking. In this study, transcriptomics was used to comprehensively analyse the protective effect of diosgenin against AIT in rats and the possible mechanism. The results showed that in the diosgenin-intervention group, compared to the model group, the expression of serum triiodothyronine, thyroxine, free triiodothyronine, and free thyroxine was decreased and that of thyroid-stimulating hormone was increased; these changes were accompanied by the downregulation of thyroglobulin, TSH receptor antibody and thyroid peroxidase expression in serum. Furthermore, transcriptome detection, RT-qPCR and immunohistochemistry verification revealed that in thyroid tissue, the relative mRNA and protein expression of cyclic adenosine 3′,5′-monophosphate (cAMP), protein kinase A (PKA) and cAMP response element-binding protein (Creb) were increased and the mRNA expression of S100 calcium-binding protein A9 (S100A9) was decreased in the diosgenin groups. In summary, diosgenin alleviates the development of AIT, possibly via the activation of the cAMP/PKA/Creb pathway and downregulation of S100A9 gene expression.

## Introduction

Autoimmune thyroiditis (AIT) is an organ-specific autoimmune disease characterized by the infiltration of immune cells, including T cells, B cells and macrophages, into thyroid tissues; the two most common types of AIT are Hashimoto’s disease (HT) and Graves’ disease (GD)^1^. HT is the most common cause of glandular atrophy and hypothyroidism, with an annual incidence rate of approximately 1 case per 1000 people^2^. In HT, a cell-mediated immune response against thyroid autoantigens (mainly thyroid peroxidase) leads to the destruction of thyroid cells^3^. GD is the most common cause of hyperthyroidism and goitre^4^, with 20 to 50 cases per 100,000 people every year^5^; it involves a humoral response against the TSH receptor, resulting in thyroid growth and hyperfunction^6^. Although the clinical manifestations of HT and GD are quite different, the two diseases are closely related. These two diseases often occur in different members of the same family, and a few individuals transition from one disease to another^7^.

Diosgenin is a well-known steroidal sapogenin and is used to treat various diseases^8^. This substance is widely found in several plants, such as *Costus speciosus*, *Trigonella foenum*, and *Smilax menispermoidea* and species of *Paris*, *Aletris*, *Trillium*, and *Dioscorea*^9^. In in vitro thyroid experiments, diosgenin intervention led to G0/G1 arrest of thyroid cells and inhibition of insulin-like growth factor-1 (IGF-1)-induced proliferation of primary human thyroid cells^10^. The ability of diosgenin to inhibit the excessive proliferation of thyroid cells may underlie its potential therapeutic effect on GD. In in vivo experiments with a murine model of Graves’ disease, diosgenin was found to reduce goitres by inhibiting the proliferation of thyroid cells. The mechanism involves the inhibition of expression of IGF-1, nuclear factor kappa-B (NF-κB), cyclin D1, and proliferating cell nuclear antigen (PCNA)^11^.

However, there has been little research regarding the effect of diosgenin on AIT, and the mechanism of its therapeutic effect on AIT is still unclear. To fully reveal the therapeutic effect of diosgenin and the underlying molecular mechanism, we detected the concentrations of serum triiodothyronine (T3), thyroxine (T4), free triiodothyronine (FT3), free thyroxine (FT4), thyroid-stimulating hormone (TSH), TSH receptor antibody (TRAb), thyroglobulin antibody (TgAb) and thyroid peroxidase antibody (TPOAb) and observed the pathological changes in thyroid tissue in vivo. Transcriptome sequencing was used to study the effect of diosgenin on whole genome expression in the thyroid tissue of AIT rats. The purpose of this study was to observe the protective effect of diosgenin in an AIT model in rats and explore the possible mechanism.

## Results

### Serum expression of thyroid function

In the AIT-model group, compared to the normal group, the serum levels of T3 (from 0.91 to 1.62 ng/ml), T4 (from 46.75 to 86.41 ng/ml), FT3 (from 1.85 to 3.57 pg/ml), and FT4 (from 3.12 to 5.61 pg/ml) were increased significantly (*P* < 0.01), whereas TSH was decreased from 4.37 µIU/ml in the normal group to 2.16 µIU/ml in the AIT-model group (*P* < 0.01). In the diosgenin high-dose group (diosgenin-H group), compared to the AIT-model group, the concentrations of T3 (from 1.62 to 1.33 ng/ml; *P* < 0.05), T4 (from 86.41 to 65.37 ng/ml; *P* < 0.01), FT3 (from 3.57 to 2.66 pg/ml; *P* < 0.01), and FT4 (from 5.61 to 4.20 pg/ml; *P* < 0.05) were decreased. TSH increased from 2.16 µIU/ml in the AIT-model group to 3.34 µIU/ml in the diosgenin-H group (*P* < 0.01) (Table 1).

**Table 1:** Comparison of serum results of thyroid function ($\bar{x} \pm SD$, n = 10).

Group	T3(ng/ml)	T4(ng/ml)	FT3(pg/ml)	FT4(pg/ml)	TSH(uIU/ml)
Normal	0.91 ± 0.19	46.75 ± 3.34	1.85 ± 0.15	3.12 ± 0.16	4.37 ± 0.63
AIT-model	1.62 ± 0.32^##^	86.41 ± 13.23^##^	3.57 ± 0.35^##^	5.61 ± 0.75^##^	2.16 ± 0.26^##^
Diosgenin-L	1.57 ± 0.28	74.40 ± 11.10	3.05 ± 0.49	4.62 ± 0.49^Δ^	2.86 ± 0.36
Diosgenin-M	1.52 ± 0.35	69.54 ± 20.05^Δ^	2.86 ± 0.44^Δ^	4.38 ± 0.62^ΔΔ^	3.29 ± 0.51^ΔΔ^
Diosgenin-H	1.33 ± 0.20^Δ^	65.37 ± 9.30^ΔΔ^	2.66 ± 0.53^ΔΔ^	4.20 ± 0.40^Δ^	3.34 ± 0.40^ΔΔ^

Note: ^#^Compared with normal group, *P* < 0.05; ^##^Compared with the normal group, *P* < 0.01; ^Δ^Compared with the model group, *P* < 0.05; ^ΔΔ^Compared with the model group, *P* < 0.01.

### Thyroid serum antibody expression

Compared to the normal group, the AIT-model group exhibited significantly increased concentrations of TgAb (from 24.30 to 147.85 IU/ml), TPOAb (from 10.56 to 168.15 IU/ml) and TRAb (from 0.94 to 24.85 IU/L) (*P* < 0.01). Compared to the AIT-model group, the diosgenin-H group exhibited decreased concentrations of TgAb (from 147.85 to 80.94 IU/ml; *P* < 0.01) and TPOAb (from 168.15 to 99.94 IU/ml; *P* < 0.01). In addition, the concentration of TRAb decreased from 24.85 IU/ml in the AIT-model group to 21.44 IU/ml in the diosgenin-M group (*P* < 0.05) (Table 2).

**Table 2:** Comparison of serum TGAb, TRAb and TPOAb expression in rats of each group.

Group	TGAb (IU/ml)	TPOAb(IU/ml)	TRAb(IU/L)
Normal	24.30 ± 4.98	10.56 ± 0.93	0.94 ± 0.30
AIT-model	147.85 ± 29.40^##^	168.15 ± 15.10^##^	24.85 ± 1.25^##^
Diosgenin-L	96.32 ± 20.15	130.33 ± 15.05	25.27 ± 3.49
Diosgenin-M	87.47 ± 21.92^Δ^	125.11 ± 16.30^Δ^	21.44 ± 3.07^Δ^
Diosgenin-H	80.94 ± 20.44^ΔΔ^	99.94 ± 10.29^ΔΔΔ^	22.15 ± 3.23

Note: ^#^Compared with normal group, *P* < 0.05; ^##^Compared with the normal group, *P* < 0.01; ^Δ^Compared with the model group, *P* < 0.05; ^ΔΔ^Compared with the model group, *P* < 0.01.

### Pathological changes in thyroid tissue

**Figure 1:**
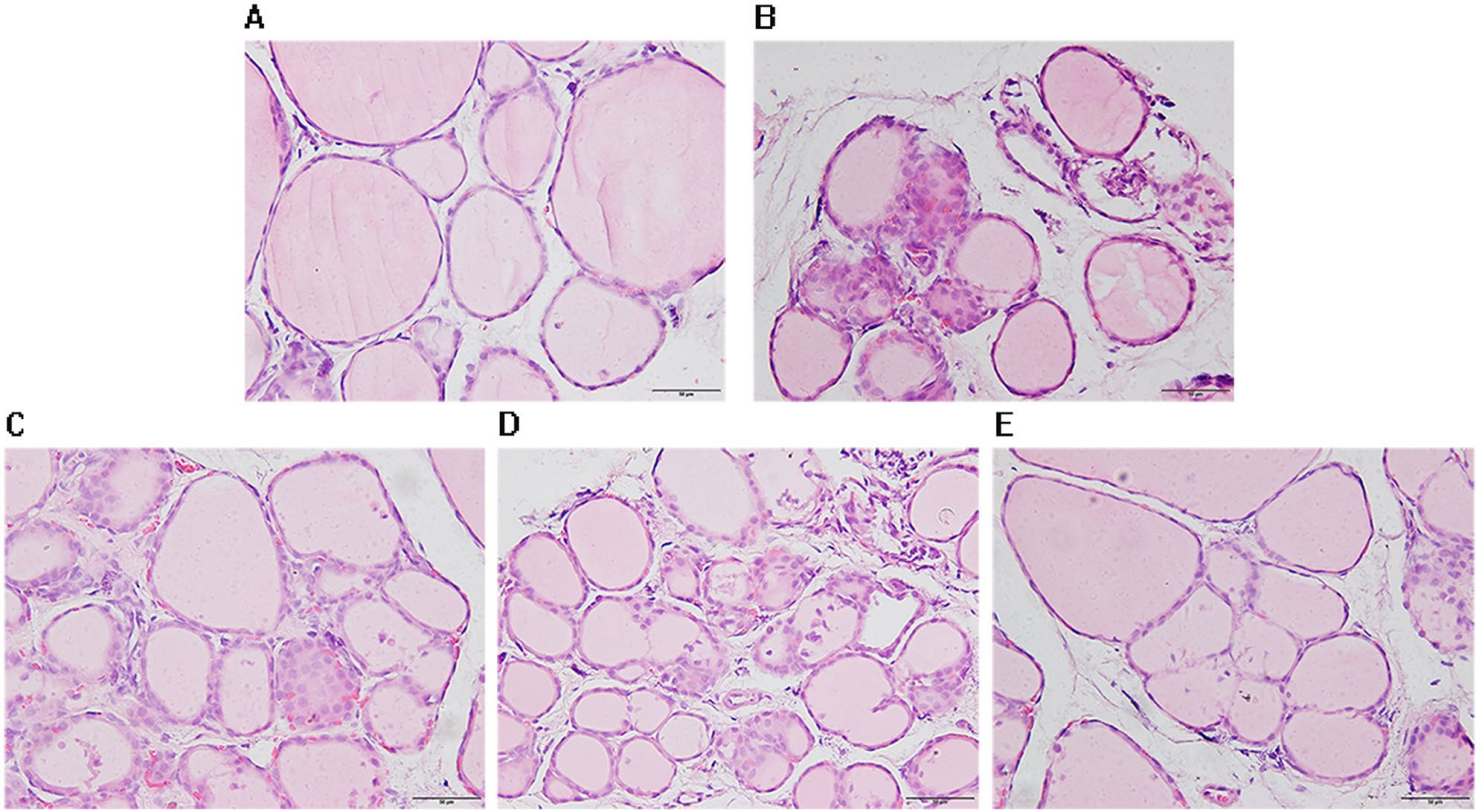
Light microscopic observation results of H&E staining of rat thyroid (× 200). (**A**) Normal group, (**B**) AIT-model group, (**C**) Diosgenin-L group, (**D**) Diosgenin-M group, (**E**): Diosgenin-H group.

A large number of intact thyroid follicles were found in the normal group. These follicles were of moderate size, were uniformly filled with red colloid, and contained intact follicular epithelial cells. In the AIT-model group, the stroma of the thyroid follicles showed diffuse lymphocyte infiltration, with a large number of follicular cavities being destroyed or reduced; the colloid within the cavities was unevenly distributed or reduced, and the follicular wall was thin or damaged. Compared to the AIT-model group, the three diosgenin treatment groups exhibited significant decreased lymphocyte infiltration into the thyroid follicular stroma, more intact follicular epithelial cells, slightly decreased colloid content, and intact follicular structure. The high-dose group had a slightly healthier profile than the low- and moderate-dose groups. See Fig. 1.

## Transcriptome

### RNA extraction and library preparation

The total RNA quality of all samples met the experimental requirements (RIN ≥ 7 and 28S/18S ≥ 0.7). The quality inspection results are shown in the supplementary materials.

### Analysis of DEGs: screening and expression analysis of differentially expressed genes

**Figure 2:**
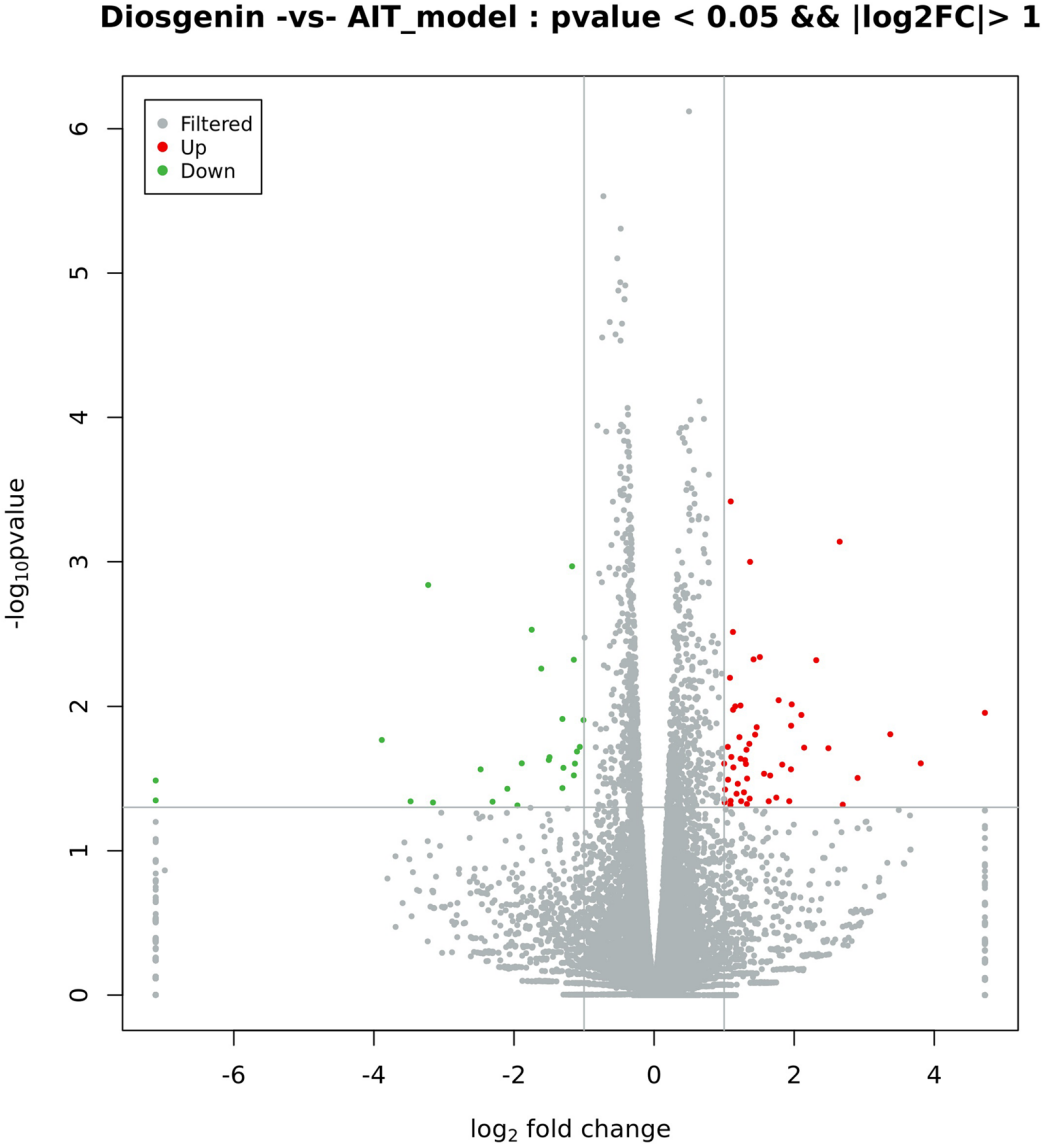
Differential expression volcano map. Photo caption: Reflection of the differences caused by comparison in the volcanic map: gray represents those genes with no significant difference, red and green represent the genes with significant differences; the x-axis displays log2FoldChange, and the y-axis displays -log10Pvalue.

**Figure 3:**
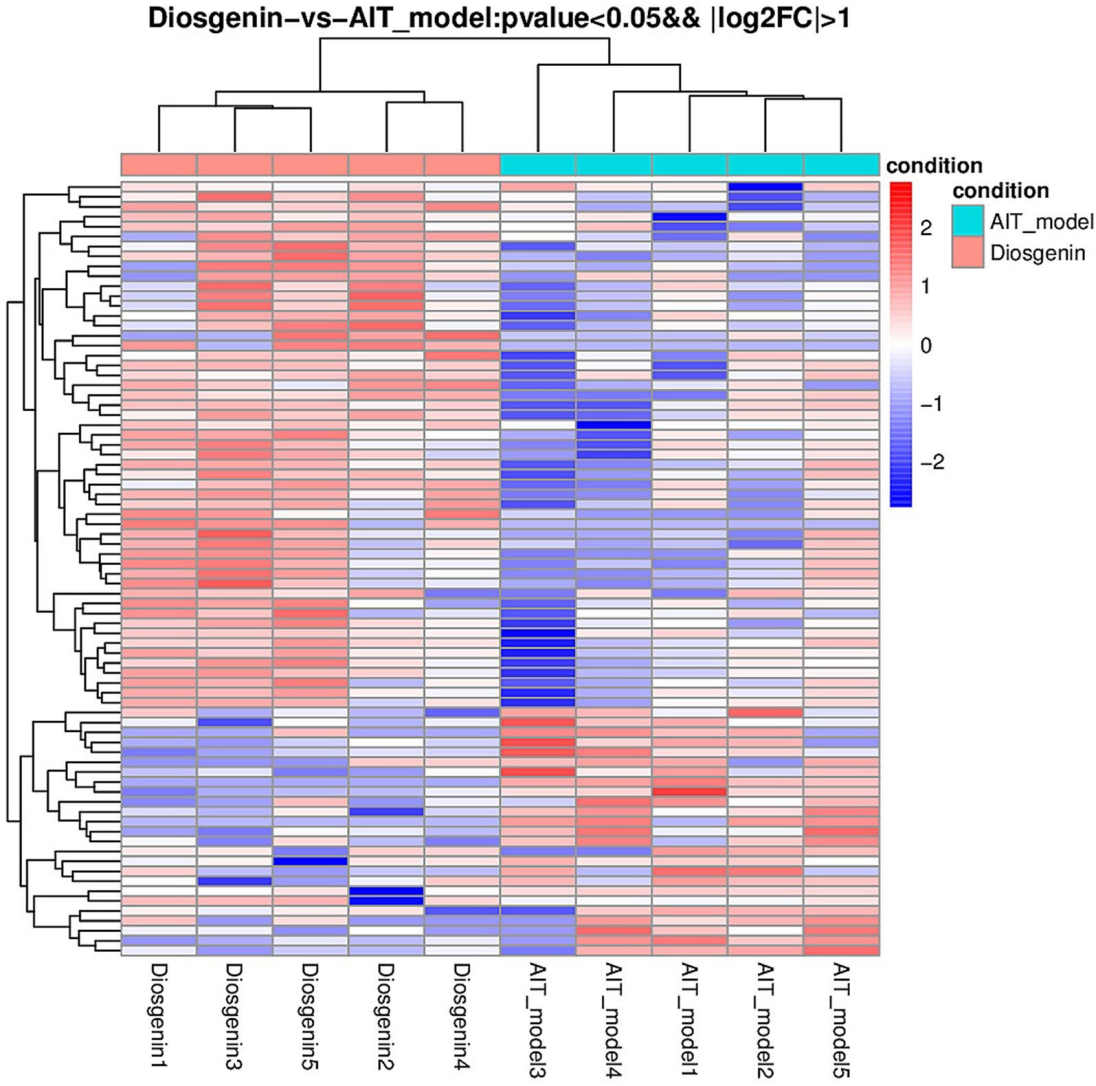
Cluster analysis results of different groups. Caption: In the picture, red indicates highly expressed genes, and blue indicates lowly expressed protein-coding genes.

DESeq software was used to analyse the number of DEGs. The number of DEGs in the diosgenin group relative to the AIT-model group was 79, comprising 54 upregulated and 25 downregulated genes. The overall distribution of DEGs was investigated by constructing a volcano plot, as shown in Fig. 2. Furthermore, we carried out unsupervised hierarchical clustering on the DEGs. Generally, the same type of samples appeared in the same clusters, and genes clustered together may have similar biological functions. See Fig. 3 for the results of the cluster analysis of differences among groups.

### GO enrichment analysis of differentially expressed genes

In the GO analysis, no differentially expressed genes related to autoimmune thyroiditis, according to GO terms, were detected among the three groups (normal, AIT-model and diosgenin groups). However, there was a differentially expressed gene associated with immune research: S100 calcium-binding protein A9 (S100A9). GO enrichment analysis showed that the expression of S100A9 in the AIT-model group was 3.509 times that in the normal group, whereas S100A9 expression in the diosgenin group was lower than that in the AIT-model group (being 0.298 times that of the AIT-model group). RT-PCR was used to verify these results.

### KEGG enrichment analysis of differentially expressed genes

**Figure 4:**
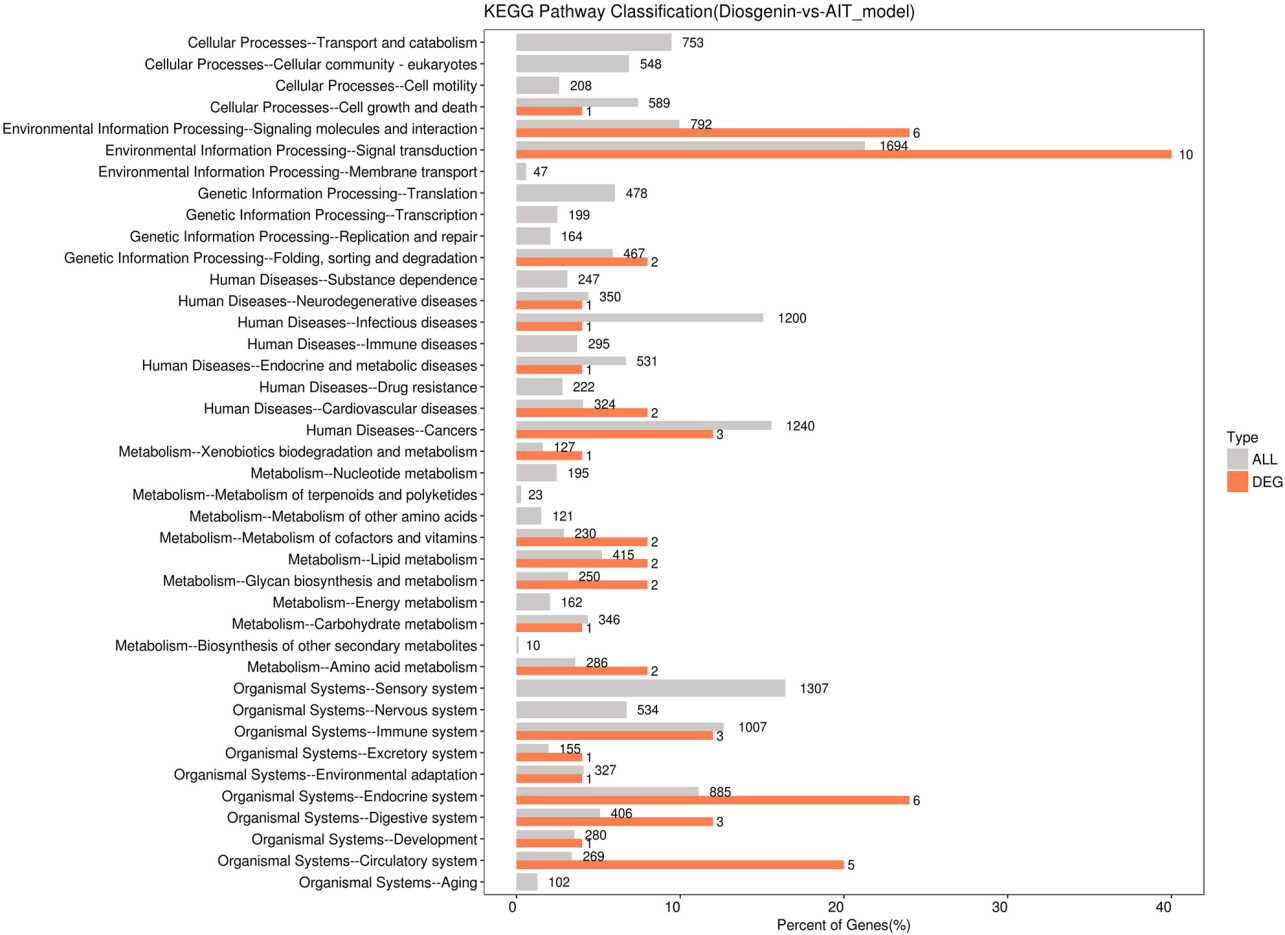
Comparison of differentially expressed genes (DEGs) and horizontal distribution of all genes in KEGG Level2. Photo caption: The horizontal axis is the ratio (%) of the total number of genes annotated to each Level2 metabolic pathway (DEGs) and all genes annotated to KEGG pathway (DEGs), the vertical axis represents the name of the Level2 pathway, and the number on the right side of the column represents the number of DEGs annotated to this Level2 pathway.

**Figure 5:**
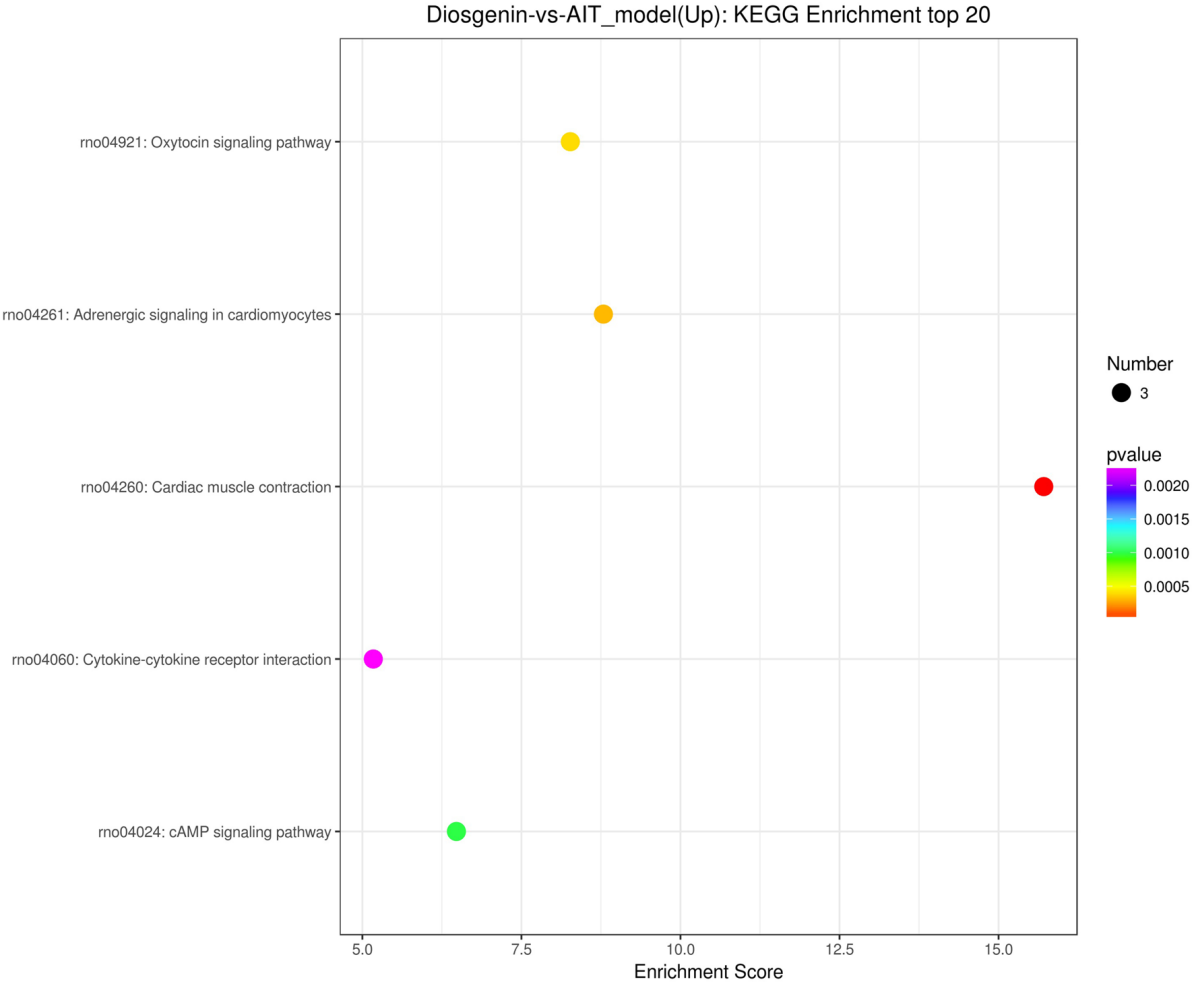
Bubble diagram of top 20 KEGG enrichment analysis pathways. Caption: The x-axis displays the enrichment score. The larger the bubble, the greater the number of differential protein-coding genes. The color of the bubble changes from purple-blue-green–red. The smaller the enrichment P-value, the greater the significance.

The level of distribution of differentially expressed genes and all genes in KEGG Level 2 in the diosgenin group, compared to those in the AIT-model group, are shown in Fig. 4. A bubble diagram of the top 20 KEGG pathways (pathway entries with > 2 differentially expressed genes were selected and sorted from largest to smallest according to − log10 *P* value) is shown in Fig. 5.

According to the disease correlation analysis of this study, the cAMP signalling pathway was selected from the top 10 pathways from the KEGG enrichment analysis (KEGG ID: rno04024, enrichment score = 6.478567553, pval = 0.000994793, padj = 0.006610834). In the subsequent experiment conducted to investigate the mechanism, we detected the expression of cyclic adenosine 3′,5′-monophosphate (cAMP), protein kinase A (PKA), cAMP response element-binding protein (Creb), exchange protein directly activated by cAMP (Epac), and Ras-related small G protein (Rap1), components in the cAMP signalling pathway.

## Real-time quantitative RT-PCR

The relative quantitative mRNA expression of Epac and Rap1 was decreased from 1 in the AIT-model group to 0.760 and 0.896, respectively, in the diosgenin-H group; the differences were not significant. PKA and Creb increased from 1 each in the AIT-model group to 2.188 and 1.536, respectively, in the diosgenin-H group (*P* < 0.05), and S100A9 decreased from 1 in the AIT-model group to 0.368 in the diosgenin-H group (*P* < 0.01) (Table 3).

**Table 3:** Comparison of PKA, Creb, Epac, Rap1 and S100A9 mRNA expression in thyroid tissue ($\bar{x} \pm SD$, n = 5).

Gene	AIT-M	Diosgenin	p-value	SD-AIT-M	SD-Diosgenin
PKA	1	2.188	0.039	0.576	1.169
Creb	1	1.536	0.033	0.306	0.381
Epac	1	0.760	0.179	0.329	0.182
Rap1	1	0.896	0.421	0.228	0.078
S100A9	1	0.368	0.001	0.268	0.071

## Immunohistochemical analysis

**Figure 6:**
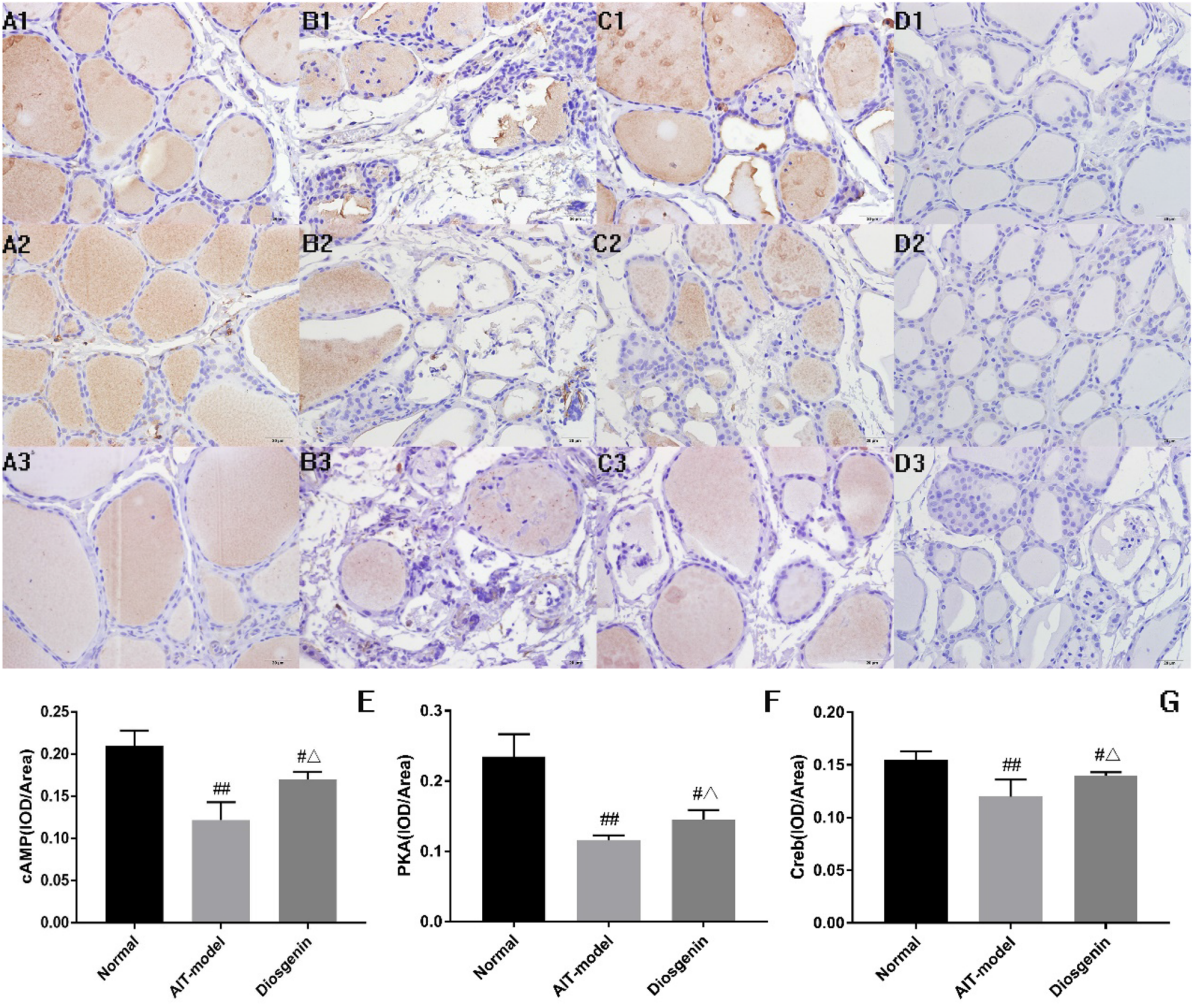
cAMP, PKA, and Creb protein levels in thyroid tissues shown by immunohistochemical staining. **A1**–**D1**: cAMP; **A2**–**D2**: PKA; **A3**–**D3**: Creb. **A1**, **A2**, **A3**: normal control; **B1**, **B2**, **B3**: AIT group; **C1**, **C2**, **C3**: Diosgenin-H; **D1**, **D2**, **D3**: Negative control staining.

Under a × 400 light microscope, the immunoreactive substances of cAMP, PKA, and Creb in thyroid tissue appeared brown and were located in the follicular stroma of the thyroid, suggesting the protein expression of cAMP, PKA, and Creb (Fig. 6). The expression of cAMP (decreasing from 0.210 to 0.122), PKA (decreasing from 0.235 to 0.116) and Creb (decreasing from 0.155 to 0.120) IOD/area in thyroid tissue of the AIT-model group rats was lower than the corresponding expression in the control group (*P* < 0.01). Compared to the model group, the diosgenin-H group exhibited increased expression of cAMP (from 0.122 to 0.170), PKA (from 0.116 to 0.145), and Creb (from 0.120 to 0.140) IOD/area (*P* < 0.05) (Table 4).

**Table 4:** cAMP, PKA and Creb IOD/Area expression in thyroid tissue ($\bar{x} \pm SD$, n = 3).

Group	cAMP	PKA	Creb
Normal	0.210 ± 0.018	0.235 ± 0.032	0.155 ± 0.008
AIT-model	0.122 ± 0.021^##^	0.116 ± 0.007^##^	0.120 ± 0.016^##^
Diosgenin-H	0.170 ± 0.009^#Δ^	0.145 ± 0.014^#Δ^	0.140 ± 0.003^#Δ^

Note: ^#^Compared with normal group, *P* < 0.05; ^##^compared with the normal group, *P* < 0.01; ^Δ^Compared with the model group, *P* < 0.05; ^ΔΔ^Compared with the model group, *P* < 0.01.

## Discussion

In this study, an AIT model was established by treating rats with high-iodine water and injecting them with porcine thyroglobulin mixed with Freund's adjuvant at multiple time points. Female Lewis rats were selected as AIT model rats because they are inbred, highly homozygous, and prone to autoimmune diseases with good repeatability^12^. The results showed that the concentrations of serum TgAb and TPOAb in the AIT-model group were significantly higher than those in the normal group (*P* < 0.01), and a large number of follicular cavities in the thyroid tissue of the AIT-model group were destroyed or reduced in size. Furthermore, the colloid content within the follicles was uneven or decreased in the AIT-model group, and this altered content was accompanied by lymphocyte infiltration. These results suggested that the AIT rat model was successfully established. Compared to those in the normal group, serum FT3, FT4 and TRAb levels in the AIT-model group were significantly increased (*P* < 0.01), whereas TSH level was decreased, meaning that this AIT rat model was biased to a hyperthyroidism model. Relative to the AIT-model group, the diosgenin low-dose and moderate-dose groups exhibited slightly improved serum T3, T4, FT3, FT4, and TSH and thyroid antibodies TgAb and TPOAb, but the differences were not statistically significant. The trends of the low-dose and moderate-dose data were similar, suggesting that the concentrations of the low-dose and moderate-dose treatments were insufficient to treat AIT. Compared to the low-dose and moderate-dose groups, the diosgenin low-dose group showed improved serum levels, and there were significant differences between the high-dose group and the model group. Furthermore, the pathology results showed that the infiltration of lymphocytes into thyroid follicular interstitial spaces was lower and the thyroid structure was less damaged in the high-dose group than in the AIT-model group. Therefore, the thyroid tissue of rats in the high-dose group was selected for transcriptome detection to explore the potential molecular mechanism underlying the protective effect of diosgenin in AIT rats.

It is difficult to carry out genetic experiments on thyroid tissue in rats or mice because the thyroid organ of rats and mice is very small and degrades easily. In addition, there is little literature on the subject. We extracted the thyroid tissues of 5 rats from each of the normal, AIT-model, and diosgenin high-dose groups and extracted the RNA, and the quality control criteria were all met. After transcriptome detection, the results were verified by RT-PCR. DESeq software was used to analyse the number of DEGs: in the diosgenin group relative to the AIT-model group, there were 79 DEGs, comprising 54 upregulated and 25 downregulated genes. These results indicated that diosgenin affected many genes with widely different functions to relieve AIT. GO enrichment analysis showed that the expression of S100A9 was increased in the AIT-model group relative to the normal group, being 3.509 times that of the normal group, whereas S100A9 expression in the diosgenin group was lower than that in the AIT-group, being 0.298 times the level of the AIT-model group. S100A9 is a member of the S100 protein family, which is involved in inflammatory reactions^13^. The S100A9 level in AIT patients has been found to be significantly higher than that in healthy controls^14^. Compared to the AIT-model group, the diosgenin-H group exhibited decreased mRNA S100A9 expression, a level 0.368 times that of the AIT-model group (*P* < 0.01), suggesting that diosgenin may slow the development of AIT by downregulating the expression of the S100A9 gene.

The differentially expressed genes were analysed by KEGG enrichment analysis, and the top 10 pathways were as follows: Cardiac muscle contraction, Adrenergic signalling in cardiomyocytes, Oxytocin signalling pathway, Glycosaminoglycan biosynthesis-keratan sulfate, Arrhythmogenic right ventricular cardiomyopathy (ARVC), TGF-beta signalling pathway, cAMP signalling pathway, Insulin secretion, and Hypertrophic cardiomyopathy (HCM). The expression results for PKA, Creb, Epac, and Rap1, components of the cAMP signalling pathway, were verified by RT-PCR. The results showed that the expression of Epac and Rap1 mRNA in the diosgenin-H group was decreased relative to that in AIT-model group but not significantly so, suggesting that cAMP might not activate Epac or Rap1 downstream. However, the expression of PKA and Creb was increased in the diosgenin-H group relative to the AIT-model group (*P* < 0.05).

Apoptosis is the main mechanism involved in thyroid cell death^15^. In Graves’ disease, there are three thyroid-stimulating hormone receptor (TSHR) antibodies with different functions (stimulation, blocking, and cleavage). The stimulating antibody can induce the survival and proliferation of thyroid cells through cAMP/PKA/Creb, whereas the cleavage antibody can induce cell death through mitochondrial ROS (mROS), and the generation of cAMP/PKA can also prevent apoptosis by inhibiting mROS^16^. The results of immunohistochemistry (IHC-P) showed that the IOD/area of cAMP/PKA/Creb in the AIT-model group was lower than that in the normal group, and there was thyroid epithelial cell injury and follicular destruction in the AIT-model group, which suggested that the low expression of cAMP/PKA/Creb was related to structural damage to the thyroid tissue. The mRNA and IOD/area of cAMP, PKA, and Creb were significantly upregulated in the diosgenin group compared to the AIT-model group, suggesting that diosgenin may induce the survival and proliferation of thyroid cells through cAMP/PKA/Creb and prevent apoptosis in thyroid cells to some degree.

Previous studies in a murine model of Graves’ disease have shown that diosgenin can inhibit the proliferation of thyroid cells by inhibiting the expression of IGF-1, NF-κB, cyclin D1, and PCNA and alleviate goitre^8,11^. The current study suggests that diosgenin may induce the survival and proliferation of thyroid cells through cAMP/PKA/Creb. Therefore, whether diosgenin can balance the proliferation of thyroid cells by inhibiting IGF-1, NF-κB, cyclin D1, and PCNA and upregulating cAMP/PKA/Creb warrants study. In addition, KEGG enrichment analysis indicates that diosgenin can significantly downregulate the expression of genes in the interleukin (IL)-17 signalling pathway. Studies^17^ have shown that Th17 and Treg cells are involved in the pathogenesis and development of AIT. Furthermore, the cellular immune balance of Th17/Treg cells plays an important role in AIT^18,19^. Therefore, whether diosgenin downregulates the expression of genes in the IL-17 signalling pathway and plays a role in Th17/Treg cellular immune balance also warrant study. In the current study, we examined only the curative effect in vivo along with its corresponding mechanism; an in vitro experiment may provide more evidence regarding the molecular mechanism of AIT.

In summary, the current study suggests that diosgenin can improve the expression of T3, T4, FT3, FT4, and TSH and decrease the concentrations of TRAb, TgAb and TPOAb in rat models of AIT. This mitigation may be related to the activation of the cAMP/PKA/Creb pathway and the downregulation of S100A9 gene expression.

## Materials and Methods

### Animals

The experiments complied with the Chinese Act on Animal Experimentation, which implements Directive 2011/588 on the Protection of Animals used for Scientific Purposes. The animal experiments were approved by the Subcommittee of Experimental Animal Ethics of the Academic Committee of Beijing University of Chinese Medicine (no. BUCM-4-2019070303-3003). The working licence number of laboratory animal practitioners was 1117052400087. All experiments were carried out in compliance with the ARRIVE guidelines.

Fifty female specific pathogen free (SPF) Lewis rats aged 4 weeks and weighing 80 ± 10 g were purchased from Beijing Vital River Laboratory Animal Technology Co., Ltd., Beijing, China; licence no. SCXK (Jing) 2016–0006.

### Reagents

Diosgenin (high performance liquid chromatography (HPLC) purity ≥ 98%, lot: C10J9Q52616, Shanghai Yuanye Bio-Technology Co., Ltd., Shanghai, China) was dissolved in 0.5% carboxymethyl cellulose sodium salt solution (lot: SL29151601, Coolaber, Beijing, China), and 0.02% dimethyl sulfoxide (lot: 1129E031, Solarbio, Beijing, China) was added to prepare the suspension for subsequent use.

Sodium iodide solution was prepared by dissolving 50 mg of sodium iodide in 100 ml of water to produce a 0.05% solution. The solution was kept away from strong light and used immediately following preparation (NaI, CAS no. 7681-82-5, 99% AR, Maya-R).

To prepare the emulsifier, a 0.1% porcine thyroglobulin (PTg) solution was prepared with phosphate-buffered saline (PBS) and then mixed with complete Freund's adjuvant (CFA) at an equal volume ratio; the mixture was then ground into an emulsion. The final concentration of PTg was 0.05%, i.e., 100 μg PTg/0.2 ml emulsifier. The preparation method of incomplete Freund’s adjuvant (IFA) was the same as that for CFA (CFA, lot: SLBW7430, Sigma, St. Louis, MO, USA; IFA, lot: SLBZ0619, Sigma; PTg, lot: 018K7012, Sigma).

### AIT induction in rats and drug treatment

#### Protocol for the AIT animal model^20^

Ten rats were randomly selected to create the normal group, and the remainder were used to establish the model. From the first week of the experiment, the model rats were free to drink 0.064% NaI, whereas the non-model rats were free to drink double distilled water. In the third week, the model rats, which had been divided into four groups, were immunized for the first time. A thyroid globulin emulsion with CFA was injected into the foot pad and subcutaneously into the back and neck of rats at multiple points (0.2 ml per injection site). Two series of injections were given, separated by an interval of 2 days. From the 4th to 8th week, the immunization was intensified: Injections of 0.2 ml into the foot pad and subcutaneously into the back and neck of the rats were performed once a week. To verify the successful establishment of the model, blood was collected from the orbital vein, and the level of TPOAb antibody in peripheral serum was detected by ELISA. Three model rats were randomly selected for observation of pathological changes in the thyroid gland.

Fifty rats were randomly divided into a normal group, an AIT-model group, a diosgenin low-dose group, a diosgenin moderate-dose group, and a diosgenin high-dose group according to their weight, with 10 rats in each group. After model establishment, the forty AIT rats were randomly divided into four groups according to their serum TPOAb value. In addition, the drug was administered. Rats in the diosgenin low-dose group (10 mg/kg.d), the diosgenin moderate-dose group (20 mg/kg.d), and the diosgenin-H group (40 mg/kg.d) were given corresponding drug suspensions, and rats in the normal group and AIT-model group were given the same volume of deionized water (1 ml/100 g.d). The treatments were continuously administered for 8 weeks.

#### Serum and pathological examination

After the treatments were completed, the rats were anaesthetized with 1% pentobarbital sodium, and blood was taken from the abdominal aorta to obtain serum. T3, T4, FT3, FT4, TSH, TRAb and TgAb were detected at the Beijing Sino-UK Institute of Biological Technology. The expression levels of TPOAb were detected by enzyme-linked immunosorbent assay (TPOAb ELISA Kit, lot: C0336030355, CUSABIO, Wuhan, China). Unilateral thyroid lobes were taken and fixed in 4% paraformaldehyde for pathological observation. The contralateral thyroid lobe was immediately placed into liquid nitrogen for transcriptome and PCR detection.

The diosgenin high-dose group was subjected to transcriptome (n = 5), RT-PCR (n = 5) and IHC-P (n = 3) analyses; the diosgenin low-dose and moderate-dose groups were excluded.

#### Transcriptome

Transcriptome sequencing and analysis were conducted by OE Biotech Co., Ltd., Shanghai, China.

#### RNA extraction and library preparation

After collection, the unilateral thyroid lobes were quickly placed in liquid nitrogen. Total RNA from thyroid lobes was extracted using a mirVana miRNA Isolation Kit (Ambion, Austin, TX, USA) following the manufacturer’s protocol. RNA integrity was evaluated using an Agilent 2100 Bioanalyzer (Agilent Technologies, Santa Clara, CA, USA). Samples with an RNA integrity number (RIN) ≥ 7 were subjected to subsequent analysis. The libraries were constructed using a TruSeq Stranded mRNA LTSample Prep Kit (Illumina, San Diego, CA, USA) according to the manufacturer’s instructions. These libraries were then sequenced on an Illumina sequencing platform (Illumina HiSeq X Ten), and 125/150 bp paired-end reads were generated.

### Bioinformatic analysis

#### Quality control and mapping

Raw data (raw reads) were processed using Trimmomatic^21^ Reads containing poly-N and low-quality reads were removed to obtain clean reads. Subsequently, the clean reads were mapped to the reference genome using HISAT2^22^.

#### Gene-level quantification, analysis of differentially expressed genes (DEGs), cluster analysis, and Gene Ontology (GO) and KEGG enrichment analyses

The fragments per kilobase of transcript per million mapped reads (FPKM)^23^ value of each gene was calculated using the Cufflinks package^24^, and the read counts of each gene were obtained using HTSeq-count^25^. DEGs were identified using the DESeq^26^ R package functions estimateSizeFactors and nbinomTest^27^. A P-value < 0.05 and fold change > 2 or fold change < 0.5 were set as thresholds for significantly different expression. Hierarchical cluster analysis of DEGs was performed to explore gene expression patterns. GO enrichment and KEGG^28^ pathway enrichment analyses of DEGs were performed using R based on the hypergeometric distribution.

#### Transcript-level quantification, analysis of differentially expressed transcripts, cluster analysis, GO, and KEGG enrichment

For transcript-level quantification, FPKM and read count values of each transcript (protein coding) were calculated using Bowtie 2^29^ and eXpress^30^. DEGs were identified using the DESeq functions estimateSizeFactors and nbinomTest. A P-value < 0.05 and fold change > 2 or fold change < 0.5 were set as the thresholds for significantly different expression. Hierarchical cluster analysis of DEGs was performed to explore transcript expression patterns. GO enrichment and KEGG pathway enrichment analyses of DEGs were performed using R based on the hypergeometric distribution.

#### Real-time quantitative RT-PCR

RNA extraction and real-time quantitative RT-PCR were conducted by OE Biotech Co., Ltd., Shanghai, China.

Quantification was performed using a two-step reaction process, employing reverse transcription (RT) and PCR. Each RT reaction consisted of 0.5 μg RNA, 2 μl of 5 × TransScript All-in-One SuperMix for qPCR and 0.5 μl of gDNA Remover in a total volume of 10 μl. Reactions were performed in a GeneAmp PCR System 9700 (Applied Biosystems, Foster City, CA, USA) for 15 min at 42 °C and subsequently for 5 s at 85 °C. Each 10 μl RT reaction mixture was then diluted × 10 in nuclease-free water and held at -20 °C.

Real-time PCR was performed using a LightCycler 480 II Real-time PCR Instrument (Roche, Basel, Switzerland) with a 10 μl PCR mixture that included 1 μl of cDNA, 5 μl of 2 × PerfectStart Green qPCR SuperMix, 0.2 μl of forward primer, 0.2 μl of reverse primer, and 3.6 μl of nuclease-free water. Reactions were conducted in a 384-well optical plate (Roche) at 94 °C for 30 s, followed by 45 cycles at 94 °C for 5 s and then 60 °C for 30 s. Each sample was run in triplicate for analysis. At the end of the PCR cycles, a melting curve analysis was performed to validate the specific generation of the expected PCR product. The primer sequences were designed in the laboratory and synthesized by TsingKe Biotech (Beijing, China) based on the mRNA sequences obtained from the National Center for Biotechnology Information (NCBI) database; the sequences are presented in Table 5. The expression levels of mRNA were normalized to that of β-actin and calculated using the 2^-ΔΔCt^ method.

**Table 5:** Primer list.

Gene	Forward primer (5 → 3)	Reverse primer (5 → 3)	Length(bp)
S100A9	GCAGCATAAGCACCATCAT	ATTTCTTTGAATTCCGCCTTG	91 bp
PKA	GGACACGAGTAACTTTGACG	AACTCAGTAAACTCCTTGCC	84 bp
Creb	CGCAGGTCCATCAGTTACA	GGATGATGAGAGCCAACGA	105 bp
Epac	CTCCGTGAGGGAAGTGAT	CCGGCAGAATTGACCTTTAC	84 bp
Rap1	CTAAGAGAGCAGATTCTTCGG	GTCTTCCAGGTCGCATTTG	81 bp
β-actin	GCGAGTACAACCTTCTTGC	TATCGTCATCCATGGCGAAC	72 bp

#### Immunohistochemistry

The main steps of immunohistochemical detection were as follows: dewaxing, hydration, 3% hydrogen peroxide treatment, high-temperature antigen repair, goat serum sealing, incubation with primary antibody at 4 °C overnight, rewarming with PBS washing, incubation with secondary antibody, diaminobenzene colour rendering, haematoxylin staining, hydrochloric acid and alcohol differentiation, tap water washing, dehydration, and sealing. Photomicrographs were taken under a microscope using an Image-Pro Plus 6.0 Media Cybernetics system to detect absorbance; analyse the integral optical density (IOD) per area of cAMP (lot: ab76238, Abcam, Cambridge, MA, USA), PKA (alpha/beta/gamma, phosphor T197, ab75991, Abcam, USA), and Creb (phosphor S133, ab32096, Abcam, USA); and carry out statistical analysis.

### Statistical analysis

SPSS v20.0 statistical software was used to process the data, which were expressed using the mean and standard deviation (x ± SD). Where the data were normally distributed and the variance was homogeneous, differences were tested by one-way ANOVA, and the LSD method was used for comparisons between groups. If the data did not conform to a normal distribution or the variance was heterogeneous, a nonparametric test was used. In addition, appropriate correction for multiple comparisons was performed. A difference was regarded statistically significant at *P* < 0.05.

## Supplementary Information

Supplementary Information

## Data Availability

The sequencing data described in this paper have been deposited in the NCBI Sequence Read Archive (PRJNA674448).
