# Supplementary material for: Transcriptome analysis of the effect of diosgenin on autoimmune thyroiditis in a rat model
Source: Sci Rep. 2021 Mar 18;11:6401. doi: 10.1038/s41598-021-85822-1 (PMC7973441; doi:10.1038/s41598-021-85822-1)
Supplement: Supplementary file 1 — Supplementary Information [file 41598_2021_85822_MOESM1_ESM.docx]

**Transcriptome Analysis of the Effect of Diosgenin on Autoimmune Thyroiditis in a Rat Model**

Chengfei Zhang^1#^, Lingling Qin^2#^, Boju Sun^1^, You Wu^1^, Fengying Zhong^1^, Lili Wu^3^, Tonghua Liu^3*^

^1^Dongfang Hospital of Beijing University of Chinese Medicine, Beijing, China

^2^Technology Department, Beijing University of Chinese Medicine, Beijing, China

^3^Key Laboratory of TCM Health Cultivation of Beijing, Beijing University of Chinese Medicine, Beijing, China

**^#^**Chengfei Zhang and Lingling Qin contribute equally to this work and co-first authors.

*** Correspondence:** Corresponding Author: Prof. Liu Tonghua. thliu@vip.163.com

RNA quality inspection results of rat thyroid tissue

| NO | Sample | concentration（μg/μl） | A260/280 | A260/230 | Volume (μl) | total（μg） | 28S/18S | RIN | score |
| --- | --- | --- | --- | --- | --- | --- | --- | --- | --- |
|  |  |  |  |  |  |  |  |  |  |
| 1 | Normal1 | 0.0343 | 1.94 | 1.89 | 10 | 0.34 | 1.6 | 8.1 | B |
| 2 | Normal2 | 0.1037 | 2.01 | 1.82 | 10 | 1.04 | 1.6 | 8.5 | A |
| 3 | Normal3 | 0.0439 | 1.88 | 1.87 | 10 | 0.44 | 1.3 | 8.6 | B |
| 4 | Normal4 | 0.0465 | 1.97 | 1.91 | 10 | 0.47 | 1.4 | 8.3 | B |
| 5 | Normal5 | 0.0411 | 2.04 | 1.81 | 10 | 0.41 | 1.4 | 8.3 | B |
| 6 | AIT-Model1 | 0.0538 | 2.00 | 1.85 | 10 | 0.54 | 1.4 | 8.3 | B |
| 7 | AIT-Model2 | 0.0318 | 1.83 | 1.85 | 10 | 0.32 | 1.6 | 8.2 | B |
| 8 | AIT-Model3 | 0.0327 | 1.91 | 1.81 | 10 | 0.33 | 1.4 | 8.4 | B |
| 9 | AIT-Model4 | 0.0637 | 2.04 | 1.85 | 10 | 0.64 | 1.5 | 8.6 | B |
| 10 | AIT-Model5 | 0.0702 | 2.09 | 1.89 | 10 | 0.70 | 1.6 | 8.7 | B |
| 11 | Diosgenin1 | 0.0928 | 2.18 | 1.89 | 25 | 2.32 | 1.5 | 8.7 | A |
| 12 | Diosgenin2 | 0.0664 | 2.06 | 1.87 | 10 | 0.66 | 1.5 | 8.6 | B |
| 13 | Diosgenin3 | 0.0764 | 2.10 | 1.80 | 10 | 0.76 | 1.5 | 8.7 | B |
| 14 | Diosgenin4 | 0.0411 | 2.00 | 1.84 | 10 | 0.41 | 1.8 | 8.7 | B |
| 15 | Diosgenin5 | 0.0826 | 2.10 | 1.85 | 10 | 0.83 | 1.5 | 8.6 | B |
